# Directed evolution of aldolases for exploitation in synthetic organic chemistry

**DOI:** 10.1016/j.abb.2008.01.005

**Published:** 2008-06-15

**Authors:** Amanda Bolt, Alan Berry, Adam Nelson

**Affiliations:** a School of Chemistry, University of Leeds, Leeds LS2 9JT, UK; b Astbury Centre for Structural Molecular Biology, University of Leeds, Leeds LS2 9JT, UK

**Keywords:** Directed evolution, Aldolases, Substrate specificity, Stereochemistry, Organic synthesis, Carbon–carbon bond formation

## Abstract

This review focuses on the directed evolution of aldolases with synthetically useful properties. Directed evolution has been used to address a number of limitations associated with the use of wild-type aldolases as catalysts in synthetic organic chemistry. The generation of aldolase enzymes with a modified or expanded substrate repertoire is described. Particular emphasis is placed on the directed evolution of aldolases with modified stereochemical properties: such enzymes can be useful catalysts in the stereoselective synthesis of biologically active small molecules. The review also describes some of the fundamental insights into mechanistic enzymology that directed evolution can provide.

The aldol reaction is a cornerstone of modern synthetic organic chemistry [1–4]. The reaction leads to the formation of a new carbon−carbon bond and up to two new stereogenic centres. The aldol reaction has, thus, been extremely widely exploited in the stereocontrolled synthesis of natural products and other complex bioactive small molecules [5]. A wide range of catalysts has been developed for the asymmetric aldol reaction including small organic molecules [6–12] (organocatalysts) and main group and transition metal coordination complexes bearing chiral ligands [13–17]. In addition, a class of enzymes—the aldolases—catalyse the asymmetric aldol reaction [18–20].

Although some enzymes with interesting aldolase activities have been obtained through rational redesign [21,22], this review will focus on the directed evolution of aldolases [23] through the evaluation of libraries of variant enzymes. We have not described interesting examples of the directed evolution of aldolases with no obvious potential for application in synthetic chemistry [24]. The many useful methods which have been developed to generate libraries of mutant proteins have been reviewed extensively elsewhere [25–27].

Directed evolution has been used to address a number of limitations associated with the use of wild-type aldolases as catalysts in synthetic organic chemistry. The generation of aldolase enzymes with a modified or expanded substrate repertoire is described. Furthermore, the directed evolution of aldolases with modified stereochemical properties is described; such enzymes can enable the synthesis of alternative, stereoisomeric products. Throughout this review, the substrates and products of aldolase-catalysed reactions are drawn in open-chain form to emphasise the catalytic differences between the wild-type and the evolved enzymes. The directed evolution of aldolases with improved thermal stability, or tolerance towards organic solvents, is, however, beyond the scope of the review [28].

There are two classes of aldolase enzymes—Class I and Class II—which are classified according to their catalytic mechanism (Fig. 1). Class I aldolases have a catalytic lysine residue which reacts with the donor substrate to yield an enamine intermediate [29–33]. The nucleophilic enamine attacks the acceptor substrate, leading to the formation of the new carbon−carbon bond; hydrolysis of the resulting imine yields the aldol product. In contrast, Class II aldolases have a catalytic zinc ion which promotes enolisation of the donor substrate [34–37]; the resulting zinc enolate intermediate attacks the acceptor substrate to yield the aldol product directly. In both cases, formation of the new carbon−carbon bond leads to the creation of up to two new stereogenic centres. The configuration of the new stereogenic centre(s) depends on which face of the aldehyde acceptor, and, in some cases, which face of the nucleophilic intermediate, reacts. Some aldolase enzymes exert exquisite control over the stereochemical course of carbon−carbon bond formation, leading to the formation of aldol products with high stereoselectivity.

A key component of any directed evolution experiment is a reliable high-throughput method for identifying variant proteins with the required properties. This can be achieved by either screening or selection. Screening involves the use of a high-throughput assay to identify mutants which catalyse the required reaction. In contrast, when selection is used, bacteria can only survive if they express a mutant protein with the required catalytic properties.

High-throughput selection methods can allow large libraries of variant aldolases to be evaluated. A variant of 2-keto-3-deoxy-6-phosphogalactonate (KDPGal) aldolase, which catalysed the formation of an alternative product, 3-deoxy-d-arabinoheptulose (DAHP)^1^, was identified using a DAHP synthase-deficient strain of *E. coli* [38,39]. Unfortunately, this general approach cannot be used to identify aldolases which catalyse the synthesis of the many interesting products which lie outside the remit of metabolism. In one such case, a screen using high-throughput gas chromatography−mass spectrometry (GC−MS) was used to assay for the production of an alternative product [40].

In many cases, it is easier to screen or select for catalysis of an aldol reaction in the reverse direction. This approach relies on the principle of microscopic reversibility: aldolases which catalyse a retro-aldol reaction must also catalyse the corresponding synthetically useful aldol condensation. The approach is valuable when one of the products of the retro-aldol reaction is a common metabolite such as pyruvate [41–46] or glyceraldehyde 3-phosphate [47]. Enzymes with the required activity can then often be identified using a screen based on a coupled enzyme assay. In addition, a screening assay has been developed in which the product of a transaldolase-catalysed reaction can spontaneously release a fluorophore [48].

*In vivo* selection methods can sometimes also be used to identify catalysts for reverse aldol reactions. For example, in one directed evolution programme, bacteria were selected on the basis of being able to utilise l-rhamnose as a sole carbon source: such bacteria were able to survive through expression of a variant aldolase able to cleave l-rhamnulose [49]. Similarly, evolved aldolases able to catalyse retro-aldol reactions yielding pyruvate have been selected using pyruvate kinase-deficient *Escherichia coli* [50].

## Directed evolution of aldolases with modified substrate specificity

Directed evolution is a powerful approach which can allow the creation of proteins with new or improved properties. The process is iterative, and proteins with the modified properties required by the researcher may be fed into subsequent rounds of evolution. The approach has been used to generate enzymes with novel, synthetically useful properties. One of the limitations of enzymes as catalysts for synthetic chemistry is that wild-type enzymes often accept only a limited range of substrates. Directed evolution has, for example, been used to broaden the synthetic scope of amine oxidases for application in the asymmetric synthesis of a wide range of amines [51–53]. Here, we describe how directed evolution has been used to expand the scope of aldolases in synthetic chemistry through modifying, or expanding, the repertoire of substrates accepted.

**Scheme 1:**
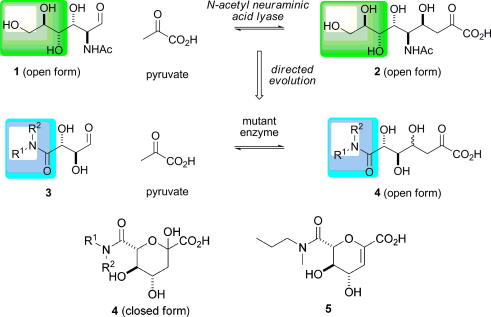
Directed evolution of an aldolase for application in the parallel synthesis of sialic acid mimetics [55]. The difference between the substrate specificity of the wild-type enzyme (green) and the E192N mutant (blue) is indicated.

*N*-Acetyl neuraminic acid lyase (NAL) is a Class I aldolase which catalyses the condensation of pyruvate with *N*-acetyl mannosamine (**1**). The wild-type enzyme accepts a wide range of five- and six-carbon aldehydes as substrates, and, thus, may be used to prepare many sialic acid derivatives. The enzyme has been evolved to increase its activity towards aldehyde substrates of general structure **3** (Scheme 1) [42]. Saturation mutagenesis was used to target residues (D191, E192, and S208) which, through structural studies [54], were known to contact the terminal triol group of a product analogue (Fig. 2). The variant aldolases were screened for their ability to catalyse the retro-aldol reaction of the substrate **4** (R^1^ = R^2^ = Pr). A variant enzyme, E192N, was identified which catalysed the cleavage of **4** (R^1^ = R^2^ = Pr) > 5-times more effectively than the wild-type enzyme catalysed the cleavage of the *N*-acetyl neuraminic acid. Remarkably, the variant enzyme E192N had broad scope, and could be exploited in the parallel synthesis of a small library of sialic acid analogues of general structure **4** [55]; related compounds, such as **5**, are potent and selective inhibitors of influenza A sialidase [56,57].

**Scheme 2:**
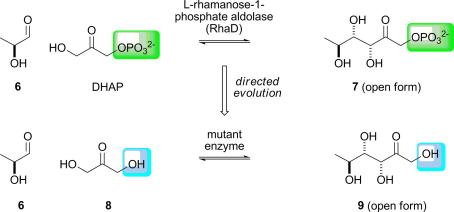
Directed evolution of l-rhamanose-1-phosphate aldolase (RhaD) to accept an unphosphorylated donor, dihydroxyacetone (**8**), as a donor [49]. The difference between substrates accepted by the wild-type (green) and the variant (blue) enzymes is indicated.

Many aldolase enzymes have evolved to accept phosphorylated substrates. However, synthetic chemists rarely want to prepare phosphorylated products, meaning that dephosphorylation after the aldol condensation is often necessary [58,59]. The problem is particularly acute with dihydroxyacetone phosphate (DHAP)-dependent aldolases which have a strict requirement for DHAP as the donor substrate. In addition, DHAP is rather unstable and, thus, can be difficult to work with. To address this problem, Wong has used directed evolution to create a variant of l-rhamulose-1-phosphate aldolase (RhaD) which accepts dihydroxyacetone (DHA,**8**) as the donor (Scheme 2) [49].

An error-prone PCR (ep-PCR) library of *rha*D genes was introduced into a selection strain, and allowed to grow on minimal media with l-rhamnose, a precursor of l-rhamnulose, as sole carbon source. The selection method involved the use of a strain of *E. coli* which was deficient in rhamnulose kinase (RhaB). These cells can only survive if the non-phosphorylated substrate, l-rhamnulose, of RhaD is accepted and cleaved by a RhaD variant to produce the glycolytic precursors l-lactaldehyde and dihydroxyacetone. Due the principle of microscopic reversibility, these variant enzymes will also be able to accept dihydroxyacetone as a substrate, and will, therefore, be useful in the synthesis of non-phosphorylated sugars. Transformants from the ep-PCR library grew after 3 days’ incubation, and the fast growing cells were enriched by seeding into a fresh minimal media/l-rhamnose broth. Two plasmids isolated from two rounds of enrichment were sequenced and were shown to possess the same two amino acid substitutions (C142Y and T158S). Hence, the use of a selection protocol allowed the directed evolution of an enzyme able to accept an unphosphorylated substrate.

**Scheme 3:**
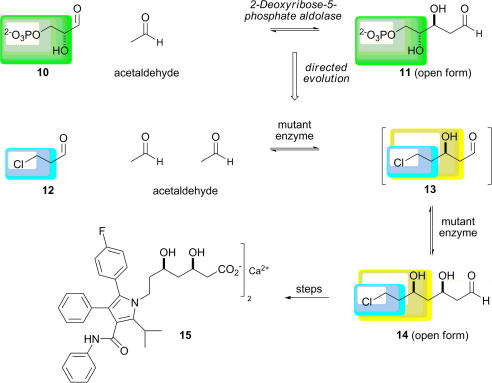
Directed evolution of 2-deoxyribose-5-phosphate aldolase (DERA) for application in the synthesis of statin drugs such as Lipitor^®^ (atorvastatin), **15**[40]. The synthesis of the aldehyde **14** involves two sequential aldolase-catalysed reactions in which 3-chloropropanal is condensed with two molecules of acetaldehyde. The variant enzyme is, thus, able to accept both of the aldehydes **12** and **13** as acceptors. The difference between the substrates accepted by the wild-type (green) and the variant (blue and yellow, for the two catalysed steps) enzymes is indicated.

2-Deoxyribose-5-phosphate aldolase (DERA) has been evolved for application in the synthesis of the statin drugs [40]. This aldolase is unusual in that it catalyses the condensation between two aldehydes: acetaldehyde and d-glyceraldehyde 3-phosphate (Scheme 3). DERA has been rationally redesigned to increase its activity towards some unphosphorylated acceptors: a mutant, S238D, exhibited a 2.5-fold improvement in activity towards d-glyceraldehyde [60,61].

Because DERA catalyses the condensation of two aldehydes, the initial condensation product can, in principle, act as an acceptor in a second condensation reaction. For example, condensation of 3-chloropropanal, **12**, with two molecules of acetaldehyde would yield a double aldol product, **14**; the aldehyde **14** is a potential precursor of the side chains of the statin drugs. 3-Chloropropanal is a poor substrate for DERA and, in fact, inactivates the enzyme at higher concentrations. A library of DERA variants was prepared using error-prone PCR. Variant enzymes were screened, using high-throughput GC–MS, for their ability to catalyse the formation of the double aldol adduct **14**. Promising variants were also assayed for resistance to inactivation by 3-chloropropanal. A C*-*terminal extended triple mutant (F200I/S258T/Y259T) had the best catalytic properties at higher concentrations of 3-chloropropanal, and was an efficient catalyst for the synthesis of the key intermediate **14**.

## General approaches for the directed evolution of aldolases for the preparation of stereoisomeric products

**Scheme 4:**
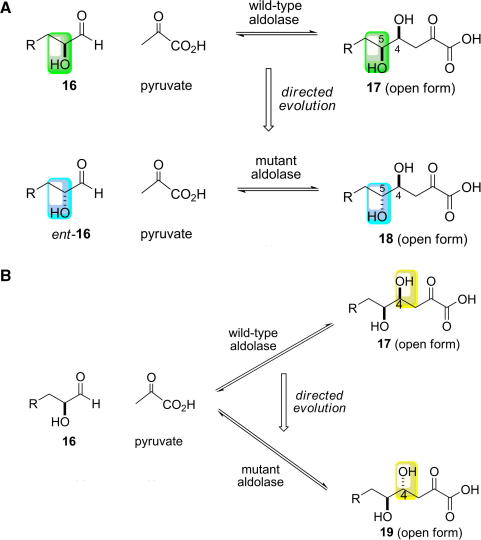
Hypothetical examples of the directed evolution of an aldolase which catalyses the condensation between pyruvate and the aldehyde **16**. (A) Directed evolution of an aldolase which accepts a stereoisomeric substrate, *ent-***16**. Here, the configuration of the newly formed stereogenic centre (C-4) is the same for the aldolase reactions catalysed by the wild-type and mutant enzymes. The products of the two reactions are diastereoisomers—**17** and **18** are epimeric at C-5, however, because the starting materials are enantiomers. The difference in the substrate specificity of the wild-type (green) and variant enzyme (blue) is indicated. (B) Directed evolution of an aldolase with a modified stereochemical course. Here, the starting materials, pyruvate and **16**, for the wild-type and mutant aldolase reactions are the same. The products are diastereoisomeric because the enzymes catalyse the attack of pyruvate on opposite faces of the aldehyde **16**, leading to the formation of stereoisomeric products**, 17** and **19**. The difference between the products is indicated in yellow.

The preparation of molecules as single stereoisomers has been a dominating theme in synthetic chemistry over the past 25 years. Many aldolase-catalysed reactions are synthetically useful, leading to products with high stereoselectivity. In many cases, however, it would be useful to be able to prepare a stereoisomer of the usual product of an aldolase-catalysed reaction. Two distinct approaches may be used to tackle this problem, both of which have been addressed using directed evolution. These alternative approaches are illustrated in Scheme 4 in the context of the directed evolution of a hypothetical pyruvate-dependent aldolase enzyme.

### Directed evolution of aldolases which accept stereoisomeric substrates

The substrate specificity of an aldolase can be altered to accept a stereoisomer of the natural substrate. This approach will inevitably lead to the formation of a product which is a diastereoisomer of the natural product (Scheme 4A). Here, the altered configuration of the product stems only from the altered configuration of the starting material accepted. For synthetic purposes, therefore, it is not strictly necessary to reverse the substrate specificity of the aldolase enzyme. An enzyme which was able to accept *both* stereoisomeric substrates could be evolved: the synthetic chemist would then be able to alter the configuration of the reaction product prepared through choice of the configuration of the substrate used.

### Directed evolution of aldolases with a modified stereochemical course

**Fig. 1:**
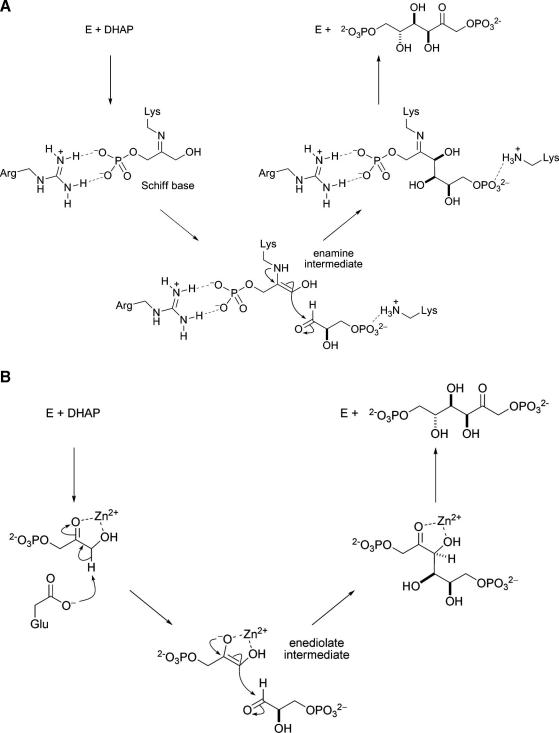
Key differences between the mechanisms of Class I and Class II aldolases illustrated for the two classes of fructose-1,6-bisphosphate (FBP) aldolase which catalyse the condensation between a donor, dihydroxyacetone phosphate (DHAP), and an acceptor, glyceraldehyde 3-phosphate. (A) In the rabbit muscle Class I aldolase (RAMA), Lys-229 has been implicated in the formation of a Schiff base with DHAP which attacks the carbonyl group of glyceraldehyde 3-phosphate [29–33]. (B) In the Class II *E. coli* FBP-aldolase, the donor DHAP is enolised by Glu-182, and the resulting enediolate intermediate attacks the acceptor substrate [34–37].

**Fig. 2:**
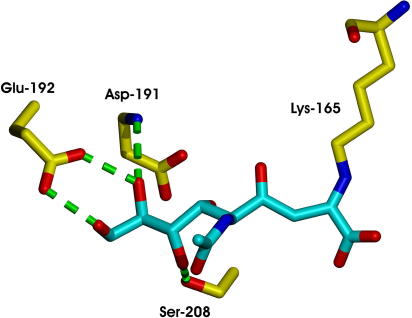
*Haemophilus influenzae* NAL structure in complex with a substrate analogue, 4-oxo-sialic acid [54], with key residues numbered according to the *E. coli* protein. The residues Asp-191, Glu-192, and Ser-208 contact the triol side chain of the substrate, and were identified as targets for saturation mutagenesis studies. Lys-165 forms a Schiff base with pyruvate.

Aldol condensation reactions can lead to the formation of up two new stereogenic centres. In the hypothetical examples in Scheme 4, the donor substrate is pyruvate and, thus, only one new stereogenic centre is formed. The configuration of the new stereogenic centre at C-4 of the products is determined by the face of the aldehyde that is attacked during the reaction mechanism (see Fig. 1). Hence, using the same substrates as the wild-type enzyme, it is possible to generate an evolved variant enzyme that could catalyse the formation of a diastereoisomeric product (Scheme 4B). Here, the altered configuration of the product stems from the modification of the stereochemical course of carbon−carbon bond formation. Unlike approach (a) (see above), a genuinely selective enzyme must be generated: the evolved enzyme must control the stereochemistry of the condensation of the substrates.

## Directed evolution of aldolases which accept stereoisomeric substrates

The preparation of stereoisomeric products of a natural aldolase reaction has now been demonstrated in a number of cases by engineering or evolving the substrate specificity of the enzyme. In each of the following examples, the enzyme was altered to accept a substrate with various structural changes including, but not confined to, its configuration. Previously, other enzymes—such as hydantoinases [62] and lipases [63]—have been evolved selectively to accept the enantiomer of the natural substrate.

**Scheme 5:**
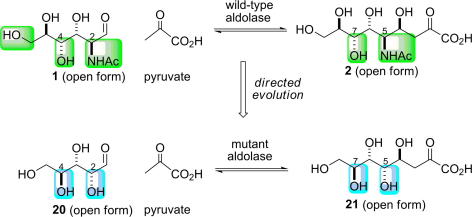
Directed evolution of *N*-acetyl neuraminic acid lyase (NAL) to accept a truncated substrate [45,46]. The differences in the substrate specificity of the wild-type (green) and variant (blue) enzymes are indicated. The substrates **1** and **20** are epimeric at both C-2 and C-4 and, hence, the products **2** and **21** are epimeric at both C-5 and C-7. In addition, **20** is a truncated analogue of **1** which also bears a different substituent at C-2.

Wong has created a variant of NAL which accepts l-arabinose (**20**) in place of *N*-acetyl mannosamine (**1**) [45,46]. The structures of l-arabinose and *N*-acetyl mannosamine differ in a number of respects. Critically, the substrates are epimeric at both C-2 and C-4, and thus the products of the enzymatic reaction would necessarily be epimeric at C-5 and C-7 (Scheme 5). In addition, however, l-arabinose is truncated by one carbon and has a hydroxyl group, rather than an *N*-acetyl group, at C-2.

Directed evolution was carried out using ep-PCR where 1000 colonies in each round were screened for their ability to accept 3-deoxy-l-*manno*-octulosonic acid (l-KDO). A coupled assay was used to monitor the activity of the mutants: upon cleavage of l-KDO, pyruvate is generated which is immediately reduced to lactate by lactate dehydrogenase (LDH), and the reaction was followed by the decrease in the fluorescent signal of NADH (excitation of 340 nm and absorbance at 450 nm). The most active mutants were selected, characterised and used as a template in subsequent rounds of ep-PCR. The best mutant was obtained after five rounds of ep-PCR: it had eight amino acid changes from the wild-type enzyme, all of which were outside the active site. This fifth-round mutant had a specificity constant (*k*_cat_/*K*_M_) for the unnatural sugar, l-KDO, which was similar to that of the wild-type enzyme for its natural substrate, *N*-acetyl d-neuraminic acid. A >1000-fold improvement in the ratio of the specificity constants [*k*_cat_/*K*_M_ (l-KDO)]/[*k*_cat_/*K*_M_ (*N*-acetyl d-neuraminic acid)] was observed.

**Scheme 6:**
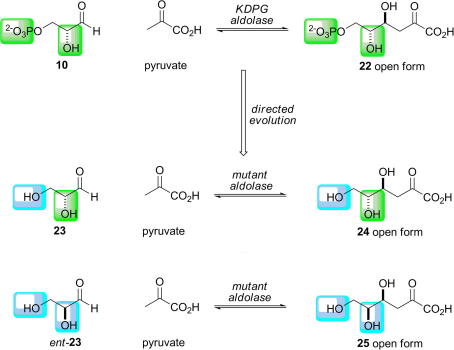
Directed evolution of d-2-keto-3-deoxy-6-phosphogluconate aldolase [43]. A variant enzyme was engineered to accept the unphosphorylated substrate, d-glyceraldehyde (**23**). However, a consequence of the evolution process was that the enantiomeric unphosphorylated substrate, l-glyceraldehyde (*ent*-**23**), was also a substrate for the variant enzyme. The variant enzyme could be used to prepare either of the diastereoisomeric products, **24** or **25**. Differences between the substrates accepted by the wild-type and variant enzymes are shown in blue and green.

Another excellent example of an aldolase able to accept a stereoisomeric substrate was also reported by Wong (Scheme 6) [43]. The first stage of the evolution of d-2-keto-3-deoxy-6-phosphogluconate (KDPG) aldolase involved the creation of an enzyme which accepted an unphosphorylated substrate. A library of 2400 KDPG-aldolase variants was created using ep-PCR with 2–3 base changes per gene. The variants were screened using a coupled enzyme assay for their ability to cleave (unphosphorylated) 2-keto-3-deoxygluconate (**24**): in this assay, 1% of the variants were more active than the wild-type enzyme. Four mutant enzymes were characterised, pooled and subjected to DNA shuffling [64]. The best second-generation variant was subjected to another round of ep-PCR, and an improved third-generation variant was identified. The third generation variant had a 3-fold increase in *k*_cat_ and a 23-fold reduction in *K*_M_, compared to the wild-type enzyme, i.e. a 70-fold improvement in catalytic efficiency (*k*_cat_/*K*_M_) towards KDG cleavage.

The ability of the third generation variant to accept both l- and d-glyceraldehyde in the synthetic direction was also investigated. d-Glyceraldehyde (**23**) is a poor substrate for the wild-type enzyme under the assay conditions, whereas the variant enzyme showed a 1.8-fold improvement in the rate of reaction. The rate of addition of pyruvate to l-glyceraldehyde (*ent-***23**) was improved >5-fold compared to the wild-type enzyme. By screening for the ability to cleave an alternative, unphosphorylated substrate, a variant KDPG aldolase was generated which was, remarkably, also able to accept the enantiomeric substrate, l-glyceraldehyde. Such an aldolase with an expanded repertoire was shown to catalyse the preparation of alternative diastereomeric products (**24** and **25**) by varying the enantiomer of glyceraldehyde supplied.

**Fig. 3:**
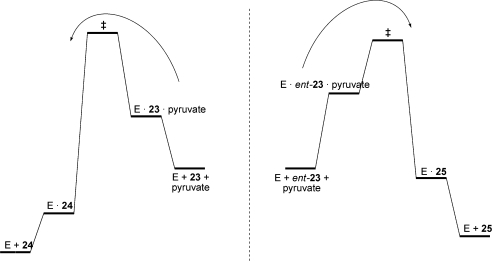
Schematic energy profile diagram of the reactions catalysed by a variant enzyme (E). The changes in Gibbs free energies schematically shown are for the concentrations of substrates used in experiments ([substrate] < *K*_M_) and not for standard states of 1 M. The outcome of the reaction is determined by the enantiomer of the substrate supplied (**23** or *ent*-**23**). The variant enzyme, E, can accept either enantiomer of glyceraldehyde as a substrate, leading to the formation of alternative products (**24** or **25**).

The implications of the catalytic properties of the evolved aldolase may be explained in terms of the schematic energy profile diagram illustrated in Fig. 3. The mutant enzyme is able to catalyse the aldol reaction of *both* enantiomers of glyceraldehyde (**23** and *ent-***23**). Thus simply by choosing the enantiomer of glyceraldehyde supplied, the stereoisomer of the product obtained can be varied.

## Directed evolution of aldolases with modified stereochemical course

The configuration of the product of an aldolase-catalysed reaction can also be varied by altering the stereochemical course of carbon−carbon bond formation. Berry and Nelson have used directed evolution to modify the stereochemical course catalysed by two different aldolase enzymes, NAL [41] and tagatose-1,6-bisphosphate aldolase (TBP-aldolase) [47].

**Fig. 4:**
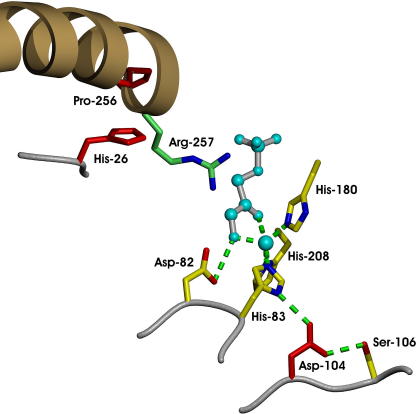
The crystal structure of tagatose-1,6-bisphosphate aldolase in complex with a DHAP analogue, phosphoglycolohydroxamate [65]. Directed evolution allowed the key role of residues 26, 104, and 256 in stereochemical control to be identified. In the wild-type enzyme, residues H26 and P256 are close to R257 which forms part of the C-6-phosphate binding site. D104 is involved in a hydrogen-bonding network with H83 which chelates the catalytic zinc ion. The H26Y/D104G/V121A/P256L variant catalysed the condensation of DHAP and d-glyceraldehyde 3-phosphate to yield fructose 1,6-bisphosphate with *ca.* 4:1 diastereoselectivity [47]. The combination of mutations identified in the variant enzyme must reorient the reactants such that, in the transition state, the *si* face of the aldehyde substrate is attacked.

**Scheme 7:**
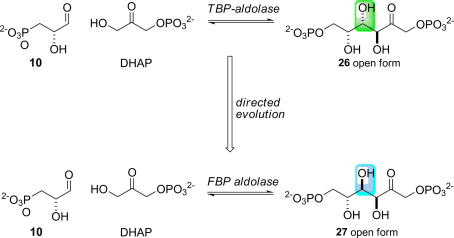
Directed evolution of tagatose-1,6-bisphosphate aldolase into a fructose 1,6-bisphosphate aldolase [47]. The stereochemical course of the reaction was altered, so that the configuration of the product at C-4 was modified. The wild-type and mutant enzymes accept the same substrates, DHAP and d-glyceraldehyde 3-phosphate (**10**), but the configuration of one of the stereogenic centres formed in the reaction is different. The differences between the structures of the products produced by the wild-type enzyme (green) and the variant enzyme (blue) are shown. The mutant enzyme produces selectively a stereoisomer, fructose 1,6-bisphosphate (**27**), of the usual product, tagatose 1,6-bisphosphate (**26**). The change in the product produced stems from attack of the enediolate intermediate derived from DHAP on the *si* face, rather than the *re* face, of the aldehyde group.

Tagatose-1,6-bisphosphate aldolase has been evolved to synthesise a (3*S*,4*S*)-configured product, fructose 1,6-bisphosphate **27**, instead of a (3*S*,4*R*)-configured product, tagatose 1,6-bisphosphate **26** (Scheme 7) [47]. Diversity was introduced into the TBP-aldolase gene through three rounds of DNA shuffling [64] with, on average, a single amino acid change per gene product. The variant enzymes were screened in the reverse direction through detection of d-glyceraldehyde 3-phosphate using a coupled enzyme assay. Surprisingly, the specificity of the evolved aldolase from the third generation of DNA shuffling (H26Y/D104G/V121A/P256L) was not further improved through saturation mutagenesis at positions 26, 104, 121, and 256. Residues His-26, Asp-104, and Pro-256 are close to the active site of the enzyme, and were identified as residues which make key contributions to stereochemical control (Fig. 4).

The evolved aldolase showed an 80-fold improvement in *k*_cat_/*K*_M_ toward the non-natural substrate, fructose 1,6-bisphosphate (**27**), resulting in a 100-fold change in stereoselectivity. The change in stereoselectivity can be attributed to attack of the donor, DHAP, on the opposite face of the acceptor aldehyde, glyceraldehyde 3-phosphate, when compared to the wild-type enzyme. It was shown, in the synthetic direction, that the mutant enzyme catalysed the formation of fructose 1,6-bisphosphate with *ca.* 4:1 diastereoselectivity.

**Scheme 8:**
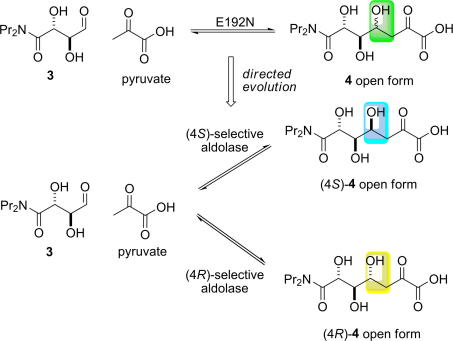
Directed evolution of a pair of complementary aldolases which catalyse aldol reactions with different stereochemical courses [41]. The reaction catalysed by the E192N variant was not very stereoselective, leading to a mixture of stereoisomeric products (green). The evolved enzymes catalyse the attack of pyruvate on opposite faces of the aldehyde **3**, leading to the selective formation of stereoisomeric products. The complementary aldolases are synthetically useful and may be used to prepare either of the products, (4*S*)- and (4*R*)-**4**, with high (>98:<2) stereoselectivity. The differences between the structures of these products are shown in blue and yellow.

The stereochemical course of a different aldolase, NAL, has also been modified by Berry and Nelson [41]. The E192N variant, previously described in this review (see Scheme 1), was shown to have broad substrate specificity. Unfortunately, the E192N variant exhibited poor stereochemical control of the aldol reaction, limiting its utility in synthetic organic chemistry. For a kinetically controlled reaction, selectivity is determined by the difference in the free energy of the competing transition states. The E192N variant catalysed the cleavage of both diastereomeric products (4*S*)- and (4*R*)-**4**. The product-determining transition states were close in free energy and, so, in the forward direction, poor stereoselectivity was observed. The E192N variant was thus used as a starting point for the creation of both (4*R*)- and (4*S*)-selective aldolases (Scheme 8).

Mutant libraries were constructed using the gene encoding E192N as the starting point. Screening was undertaken in the reverse aldol direction, with detection of pyruvate using a coupled enzyme assay. Promising mutants were probed using both diastereoisomeric screening substrates (4*S*)- and (4*R*)-**4** in order that genuinely selective enzymes were identified.

**Fig. 5:**
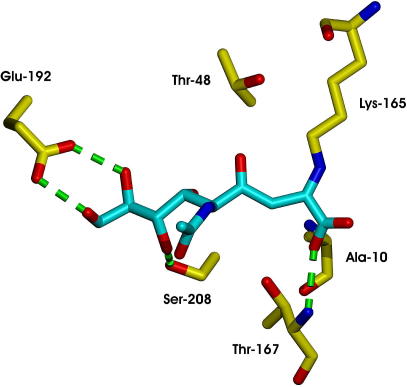
*Haemophilus influenzae* NAL structure in complex with a substrate analogue, 4-oxo-sialic acid [54], with key residues numbered according to the *E. coli* protein. Residues Ala-10, Thr-48, Ser-208, and Thr-167 are highlighted and have been shown to play a key role in controlling the stereochemical course of carbon−carbon bond formation. Lys-165 forms a Schiff base with pyruvate. The variants E192N/T167V and E192N/T167G were selective for the cleavage of (4*R*)- and (4*S*)-**4**, respectively, and, thus, residue 167 plays a particularly important role in stereochemical control. From the programme as a whole, E192N/T167V/S208V was most selective for the cleavage of (4*R*)-**4**, whereas E192N/T167G was most selective for the cleavage of (4*S*)-**4**.

In the first round of directed evolution, an ep-PCR library was constructed, and ∼2500 clones were screened. About 1% of library members showed significant differences in activity between the two diastereoisomeric screening substrates, (4*S*)- and (4*R*)-**4**. Two variants were selected as displaying greater stereoselectivity than the E192N ‘parental’ variant. The chosen 4*R*-selective mutant contained four changes in addition to the E192N substitution: A93V, F109I, N153K, and S208G. In contrast, the chosen 4*S*-selective variant contained a different set of six substitutions (compared to the E192N ‘parent’): T3M, A10V, T48A, Q61R, E64D, and Q173R. Site-directed mutagenesis was used to identify which of these residues play a dominant role in stereochemical control. Three key residues, Ala-10 and Thr-48 and Ser-208 (*E. coli* numbering), all of which make direct contact with the substrate, were found to contribute to controlling the stereochemistry of carbon−carbon bond formation (Fig. 5). The mutations T48V and A10V were found to act synergistically, and combined—in A10V/T48V/E192N—to create an enzyme which was selective for cleavage of (4*S*)-**4**.

The results of screening the ep-PCR library guided further experiments. In addition, residue 167 was identified as a potentially important residue since the equivalent residue, Thr-157, in 2-keto-3-deoxygluconate aldolase (KDGA) makes a hydrogen bond to the C-4 hydroxyl group of its product [66]. Saturation mutagenesis of the amino acids in positions 10, 48, 167, and 208 was, therefore, undertaken to optimise the stereochemical properties of the aldolases. The E192N/T167V and E192N/S208V variants were found to be selective for the cleavage of (4*R*)-**4**. In contrast, E192N/T167G showed excellent stereochemical discrimination, and was, at least 50-fold selective for cleavage of the diastereoisomeric substrate (4*S*)-**4**. These experiments showed that the variants E192N/T167V and E192N/T167G had complementary catalytic properties and, thus, that residue 167 plays a particularly important role in stereochemical control. The most 4*S*-selective enzyme, E_4_*_S_*, was the double mutant E192N/T167G. The most 4*R*-selective enzyme, the triple mutant, E_4_*_R_* (T167V/E192N/S208V), was discovered by combining key mutations (T167V and S208V) identified in earlier generations.

The most 4*S*- and 4*R*-selective aldolases were shown to be useful catalysts for synthetic chemistry: condensation of pyruvate with the aldehyde (**3**) allowed either stereoisomeric product—(4*S*)- or (4*R*)-**4**—to be prepared simply by choosing the enzyme—E_4_*_S_* or E_4_*_R_*—used.

**Fig. 6:**
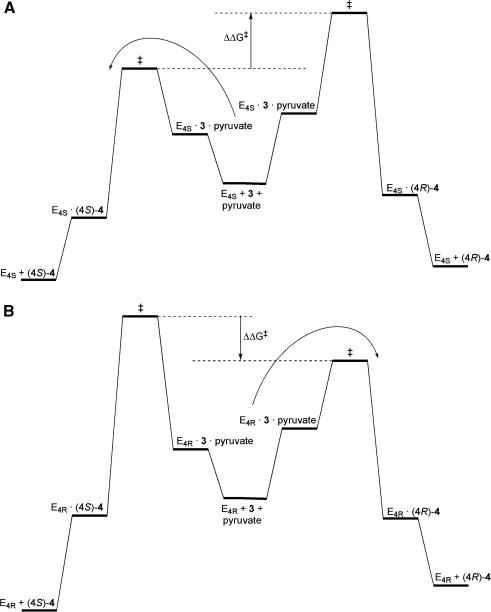
Engineering the stereochemical course of aldolase reactions. (A and B) Schematic energy profile diagrams of the complementary 4*S*- and 4*R*-selective aldolases, E_4_*_S_* and E_4_*_R_*. The changes in Gibbs free energies schematically shown are for the concentrations of substrates used in experiments ([substrate] < *K*_M_) and not for standard states of 1 M. In each case, the enzyme determines the configuration of stereogenic centres formed in the aldol reaction. The starting materials (e.g. pyruvate and **3**) may condense to yield two possible stereoisomeric products, (4*S*)- or (4*R*)-**4**. In order for the reaction to be synthetically useful, the enzymes must control the stereochemical course of the reaction such that only one stereoisomer is produced with high stereoselectivity. The stereochemical course is controlled by the difference in the free energy (ΔΔ*G*^‡^) between the two product-determining transition states. A pair of stereoselective variants of NAL was created, E_4_*_S_* and E_4_*_R_*, allowing both of the possible stereoisomeric products, (4*S*)- and (4*R*)-**4**, to be prepared with high (>98:<2) stereoselectivity: the product obtained simply depended on the variant enzyme (E_4_*_S_* and E_4_*_R_*) used.

The outcome of the directed evolution of a pair of stereochemically complementary aldolases is illustrated schematically in Fig. 6. Earlier in this review, we described an example of a variant aldolase which was able to accept *either* enantiomer of glyceraldehyde as a substrate, and the choice of which stereoisomer was prepared was determined by varying which substrate was supplied. In contrast, highly selective enzymes are required to control the stereochemical course of carbon−carbon bond formation. Here, the same substrates can condense to give alternative stereoisomeric products, and the enzyme must control this process in order that single stereoisomers are produced. Both of the complementary NAL variants control the stereochemical course of carbon−carbon bond formation: in both cases, there is a significant difference in free energy (ΔΔ*G*^‡^) between the product-determining transitions states. The choice from a pair of variant enzymes can, thus, be used to choose to prepare either (4*S*)- and (4*R*)-**4** with high stereoselectivity.

A fascinating outcome of directed evolution is that new insights into mechanistic enzymology can be gained. In the evolution of the stereochemical course of two aldolases—NAL and tagatose-1,6-bisphosphate aldolase—the residues responsible for stereocontrol were close to the enzyme active site. These residues must steer the reaction between the donor and acceptor substrates, controlling the face of the aldehyde that is attacked. The discovery—for two aldolases—that a small number of residues close to the active site can control the stereochemical course of carbon−carbon bond formation is remarkable, and may help guide other directed evolution programmes. With NAL, the key residues identified were all in the active site of the enzyme. In contrast, with tagatose-1,6-bisphosphate aldolase, the key residues were slightly more remote, and perturbed hydrogen bonding networks with the substrate. By targeting residues close to the active site, it may, however, be possible to modify the stereochemical course of many other carbon−carbon bond-forming enzymes. Such enzymes would also be of huge value in stereocontrolled organic synthesis.

## Summary

In this review, we have focussed on the directed evolution of aldolases with synthetically useful properties. To start with, we described examples of evolved aldolases with broadened substrate specificities. Such enzymes are useful catalysts for synthetic chemistry since they catalyse the formation of unnatural products: products which are no longer phosphorylated, or which have modified side chains. The directed evolution of enzymes with these novel activities can provide new insights into the factors which allow enzymes to recognise substrates and transition states.

In the second part of the review, we described how directed evolution may be used to create enzymes for stereoselective organic synthesis. Promiscuous aldolases can be created which are able to accept more than one stereoisomeric substrate: such aldolases can be used to prepare more than one stereoisomeric product through choice of the substrate provided. It is also possible to create enzymes with modified stereochemical courses, that is enzymes which control the configuration of stereogenic centres formed in the aldol reaction. In order to be synthetically useful, such enzymes need to be highly stereoselective, thus determining the stereoisomer prepared. The evolution of enzymes with modified stereochemical courses can provide new insights into the mechanistic enzymology. Such insights might be useful in the directed evolution of other stereoselective carbon−carbon bond-forming enzymes.

## Footnotes

1 *Abbreviations used:* DAHP, 3-deoxy-d-arabinoheptulose; GC−MS, gas chromatography−mass spectrometry; DHAP, dihydroxyacetone phosphate; RhaD, l-rhamulose-1-phosphate aldolase.
